# Evaluation of functional, structural, and electrophysiological optic nerve changes following extradural anterior clinoidectomy in patients without preoperative optic canal compression

**DOI:** 10.1007/s10143-026-04320-y

**Published:** 2026-05-09

**Authors:** Mohammed Aladdam, Mehmet Sabri Gürbüz, Gülşen İshakoğlu, Fehim Esen, Temel Tombul, Fatih Çalış

**Affiliations:** 1 Department of Neurosurgery, Göztepe Prof. Dr. Süleyman Yalçın City Hospital, Istanbul Medeniyet University, Eğitim Mah. Fahrettin Kerim Gökay Caddesi Kadıköy, Istanbul, 34722 Türkiye; 2 Department of Ophthalmology, Göztepe Prof. Dr. Süleyman Yalçın City Hospital, Istanbul Medeniyet University, Istanbul, Türkiye; 3 Department of Neurology, Göztepe Prof. Dr. Süleyman Yalçın City Hospital, Istanbul Medeniyet University, Istanbul, Türkiye

**Keywords:** Extradural anterior clinoidectomy, Optic nerve, Optic canal unroofing, RNFL, Visual evoked potentials, Neurovascular surgery

## Abstract

Extradural anterior clinoidectomy is increasingly used to improve exposure and proximal vascular control in aneurysm surgery, but its isolated effect on an otherwise uncompromised optic nerve remains unclear. To evaluate the functional, structural, and electrophysiological impact of EAC with optic canal unroofing in patients without preoperative optic nerve compression or optic canal pathology. We conducted this single-center study included 16 adults who underwent no-drill extradural anterior clinoidectomy (EAC) during microsurgical clipping of ruptured aneurysms (January 2023–December 2024). Patients with visual or optic pathway pathology were excluded. Postoperative assessment (6–12 months) included visual acuity, automated perimetry (visual field index, VFI), OCT–derived retinal nerve fiber layer (RNFL) thickness, and visual evoked potentials (P100 latency). Eyes were compared using paired tests, with repeated-measures ANOVA for quadrant-based ΔRNFL. Visual acuity was preserved in all patients. Global RNFL thickness was similar in ipsilateral and contralateral eyes (95.8 ± 12.7 vs. 99.2 ± 18.6 μm; p = 0.230). Quadrant ΔRNFL varied by quadrant, but no pairwise differences remained after correction, with a trend toward greater thinning in the superior quadrant. VFI was similar (p = 0.7); one patient had inferior nasal quadrantanopia and two had mild blind-spot enlargement. P100 latency was comparable (114.8 ± 9.7 ms vs. 113.8 ± 8.9 ms; p = 0.223). No major EAC-related neurovascular complications were observed. Extradural anterior clinoidectomy was not associated with statistically significant optic nerve impairment, although visual field changes occurred in 3/16 patients (18.75%) with a trend toward superior RNFL thinning.

## Introduction

Anterior clinoidectomy was first described in 1952 by Hauser and Gass as a neurosurgical technique for decompression of the optic nerve in a patient who presented with visual loss secondary to an internal carotid artery aneurysm; the authors originally proposed an intradural approach [8]. Hakuba described the technique of EAC first in his article in Japanese in 1982 which he had used in 19 patients of different pathologies [7]. Later, in 1985, Dolenc popularized the extradural technique and it gained wide acceptance thereafter [6]. Initially, this approach was used mainly to facilitate access to carotico-ophthalmic aneurysms and to achieve optic nerve decompression. With advances in microsurgical techniques and a better understanding of skull base anatomy, the extradural approach gained wider acceptance. The technique popularized by Dolenc allows safer removal of the anterior clinoid process before dural opening and reduces the risk of injury to neurovascular structures closely related to the anterior clinoid process, such as the optic nerve, the clinoidal segment of the internal carotid artery, and the oculomotor nerve [11, 13].

Traditionally, anterior clinoidectomy has been performed in cases with optic nerve compression to decompress the nerve and improve visual function, as well as to achieve early devascularization of meningiomas [9]. However, in recent years, its use has not been limited to decompression alone; it has increasingly been employed to obtain better access to the optic nerve, optic chiasm, and to gain better proximal control in the anterior and posterior cerebral vascular systems [5, 15]. This shift in surgical practice reflects the increasing familiarity and growing adoption of the procedure to facilitate safer and more effective access to critical neurovascular structures. Anterior clinoidectomy is now most commonly employed in the treatment of vascular and neoplastic lesions of the skull base. In the vascular domain, it facilitates proximal control and improved exposure for ophthalmic, paraclinoid, posterior communicating, and superior hypophyseal artery aneurysms, and is also used in selected basilar apex aneurysms [10]. Among tumors, anterior clinoidectomy improves safe resection and surgical access for clinoidal and suprasellar meningiomas, pituitary adenomas, craniopharyngiomas, chordomas, and sinonasal malignancies with anterior clinoid extension [9].

In our series, extradural anterior clinoidectomy was selectively performed in ruptured aneurysm cases in which early cisternal access was limited due to brain tightness. In these situations, limited removal of the anterior clinoid process provided additional exposure to the opticocarotid and chiasmatic cisterns, facilitating earlier cerebrospinal fluid release and reducing the need for brain rectraction.

The optic nerve occupies a critical position at the skull base and traverses the optic canal, which courses inferomedially to the anterior clinoid process. Because of this intimate anatomic relationship, extradural anterior clinoidectomy (EAC) inherently carries certain risks for the optic nerve. Potential mechanisms of injury include direct trauma during placement of micro-rongeurs into the optic canal, thermal damage generated by high-speed drilling, and inadvertent traction transmitted during bone removal. Furthermore, the ophthalmic artery, which often arises near the clinoidal segment of the internal carotid artery, may be injured during the procedure, thereby increasing the risk of ischemic optic neuropathy [1, 10]. Importantly, even in patients without preoperative optic nerve compression, the possibility of iatrogenic visual loss must be considered. To the best of our knowledge, there are no studies in the literature that directly evaluate the impact of EAC on optic nerve function in cases without preexisting optic canal compression.

Most existing reports on EAC focus on cases where tumors or aneurysms invade the optic canal or cause visual impairment, and visual outcomes are often attributed primarily to the underlying pathology rather than the clinoidectomy itself [3, 11]. Moreover, many studies rely on crude measures such as Snellen acuity or non-standardized visual field assessment, with limited use of multimodal quantitative testing. As a result, the isolated effect of EAC and optic canal unroofing on an otherwise uncompromised optic nerve remains poorly defined.

The aim of the present study is to evaluate the structural, functional, and electrophysiological effects of extradural anterior clinoidectomy performed solely to enhance surgical exposure in patients without preoperative optic nerve compression or optic canal stenosis. To this end, we used a multimodal assessment strategy that included best-corrected visual acuity, automated perimetry (Visual Field Index), optical coherence tomography (OCT)–derived retinal nerve fiber layer (RNFL) thickness, and visual evoked potentials (VEP).

## Materials & methods

### Study design

This single-center retrospective study was conducted at İstanbul Medeniyet University Göztepe Prof. Dr. Süleyman Yalçın City Hospital. All patients who underwent extradural anterior clinoidectomy (EAC) between 1 January 2023 and 1 December 2024 and met the predefined inclusion criteria were eligible for analysis. The study protocol was approved by the Institutional Review Board of Istanbul Medeniyet University (2025/09–21), and all procedures were carried out in accordance with the ethical standards of the Declaration of Helsinki. To minimize potential confounders related to early postoperative recovery and delayed visual pathway changes, comprehensive optic nerve function assessments were performed between 6 and 12 months after surgery.

### Inclusion criteria

Patients were eligible if they met all the following:

- Age ≥ 18 years.
- Glasgow Coma Scale (GCS) score of 15 with adequate orientation and cooperation to complete all optic nerve function tests reliably.
- Underwent extradural anterior clinoidectomy performed solely to improve surgical exposure/early CSF drainage during microsurgical clipping of a ruptured intracranial aneurysm.
- Completed the full multimodal ophthalmologic and electrophysiological assessment protocol at the planned postoperative time point.

### Exclusion criteria

Patients were excluded if any of the following were present:

- Preoperative visual complaints or a documented visual deficit.
- Known ophthalmologic or neurological disease that could affect vision.
- Radiological or intraoperative evidence of optic nerve, optic canal, or chiasmal compression by an adjacent aneurysm or other lesion.
- Pathologies directly involving the optic nerve or optic canal (e.g., tuberculum sellae meningioma, optic nerve sheath meningioma, sphenoid wing/sphenoorbital meningiomas).
- Marked clinoid or paraclinoid pneumatization, or other major anatomic variants of the clinoid region that altered the standard EAC procedure.
- Previous orbital or optic canal surgery.
- Paraclinoid internal carotid artery aneurysms regardless of size (due to possible optic nerve impingement).
- Large anterior communicating artery aneurysms regardless of orientation (Acomm aneurysm > 10 mm were excluded).
- The use of three or more aneurysms in the same procedure, because the cumulative clip bulk could compress the optic apparatus and confound effects attributable to the clinoidectomy itself.

Posterior communicating artery aneurysms were not excluded unless there was clear radiological or intraoperative evidence of compression of the optic pathways.

### Anterior clinoid process anatomy

The anterior clinoid process (ACP) is a bony projection forming the posterior and medial portion of the lesser wing of the sphenoid. It is a pyramidal structure with three main bony attachments: anterolaterally, it continues as an extension of the lesser wing; anteromedially, it forms the roof of the optic canal and contributes to the planum sphenoidale; and posteroinferiorly, it joins the body of the sphenoid via the optic strut, a thin bony bar that separates the optic canal from the superior orbital fissure. Medially, the ACP lies in close relationship to the internal carotid artery and the optic nerve, while inferiorly it neighbors the superior orbital fissure and cavernous sinus; among the neurovascular structures in this region, the oculomotor nerve is the closest inferior structure [2, 9, 16].

## Surgical technique

All procedures were performed by a senior neurosurgeon (M.S.G) at our institution using a standard pterional craniotomy approach with interfascial temporalis flap. In patients who met the inclusion and exclusion criteria, extradural anterior clinoidectomy (EAC) was performed to facilitate exposure of the basal cisterns and early cerebrospinal fluid (CSF) release. Although this technique is not widely used for anterior communicating artery aneurysms, the senior author prefers to employ it selectively to improve early visualization of the opticocarotid cisterns and as an adjunct to lamina terminalis opening for early brain relaxation in ruptured ACom aneurysm surgery. In our patients no-drill extradural anterior clinoidectomy technique was used, with removal of the anterior clinoid process performed using bone rongeurs and microsurgical instruments.

**Fig. 1:**
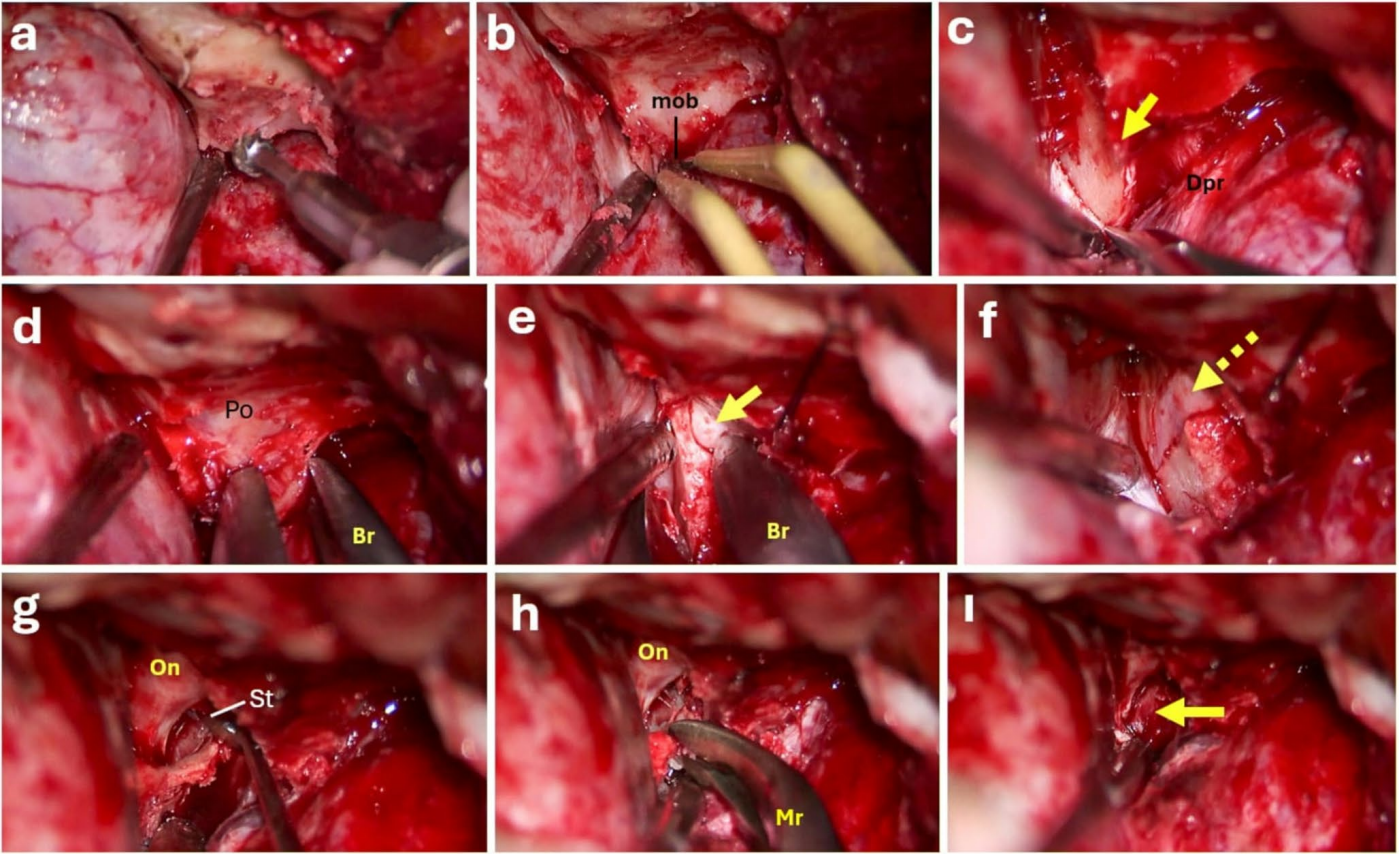
Intraoperative photographs illustrating the stepwise no-drill extradural anterior clinoidectomy (**a**) Extradural drilling of the sphenoid wing until the meningoorbital band is confronted. (**b**) Coagulation and division of the meningo-orbital (MOB) band following flattening of the sphenoid ridge and orbital roof (**c**) Dissection of the temporal dura propria (Dpr) from the periosteal dura of the lateral wall of the cavernous sinus to expose the anterior clinoid process along its entire length; the yellow arrow indicates the divided MOB. (**d**) Opening the lateral orbital wall with a drill which is the only drilling point in this technique; Po = periorbita; Br = baby rongeur. (**e**) Through the orbital roof opening, a baby rongeur and Lempert micro-rongeur are advanced along the periorbita laterally/anteriorly toward the optic canal medially/posteriorly, severing the superomedial attachment of the anterior clinoid process; the arrow denotes the optic nerve entry into the periorbita. (**f**) Optic nerve is totally exposed following unroofing of the optic canal (arrow). (**g**) Following careful biting and removal of the optic strut (St) piecemeal removal of the anterior clinoid process is achieved, exposing the optic nerve (ON); a Rhoton dissector is used to release residual soft-tissue attachments to facilitate removal. (**h**) A micro-rongeur (Mr) is used to trim the optic strut and carefully mobilize the anterior clinoid process. (**i**) Final clinoid space after complete resection. Small communications between the clinoid space and the cavernous sinus may result in mild venous oozing, which is readily controlled with hemostatic agents

In the present study, the term “no-drill” specifically refers to the clinoidectomy phase. A high-speed drill was used only during the preparatory exposure phase to thin and open the lateral orbital wall and flatten the sphenoid ridge, as illustrated in Fig. 1d. No drilling was applied directly to the anterior clinoid process, optic canal roof, or optic strut. Removal of the anterior clinoid process and optic canal roof was performed exclusively using micro-rongeurs and Kerrison instruments. Accordingly, this approach may be more precisely described as a partial no-drill clinoidectomy, as drilling is limited to the exposure phase and not the clinoidectomy itself.

Following completion of the pterional craniotomy and elevation of the bone flap, the sphenoid wing was removed using a combination of high-speed drill and bone rongeurs. The posterolateral portion of the orbital roof was then opened using high-speed drill to expose the periorbita. The periorbita was carefully dissected from the orbital roof using a fine dental instrument.

Subsequently, the lateral orbital wall was progressively thinned and removed using bone rongeurs. The meningo-orbital band was identified and sharply transected using a No-11 surgical blade in a direction parallel to the superior orbital fissure. This allowed for identification and separation of the surgical dissection plane between the dura propria and the dural over the lateral wall of the cavernous sinus.

The optic canal was then carefully unroofed using a micro-rongeur, and the optic strut was removed using a combination of microrongeur and 1 mm Kerrison rongeur. Finally, the anterior clinoid process was removed in a piecemeal fashion using a micro-rongeur.

Hemostasis during sphenoid wing and clinoid process removal was initially achieved using bone wax. Following removal of the anterior clinoid process, gelatin–thrombin hemostatic matrix was applied to the clinoid space to control venous bleeding. In cases of persistent venous oozing from the clinoid space additional hemostatic agents like fibrin glue were used to maintain a clear operative field (Fig. 1).

### Optic nerve evaluation protocol

Comprehensive postoperative assessment of optic nerve function was performed between 6- and 12-months following surgery to evaluate long-term functional, micro-structural and electrophysiological integrity of the optic nerve postoperatively. The evaluation consisted of four standardized tests described below:

1. Visual Acuity (VA)
  Best-corrected visual acuity was assessed using a standard Snellen chart under controlled lighting conditions. The test was conducted monocularly for each eye, and results were recorded as Snellen fractions and converted to decimal format for analysis. Only best-corrected values were included to ensure the exclusion of refractive errors as a source of visual impairment.
2. Visual Field (VF)
  Automated visual field testing was performed using the Humphrey Field Analyzer (Carl Zeiss Meditec, Dublin, CA) with the SITA Standard 24 − 2 protocol, which evaluates 54 test points within the central 24 degrees of the visual field. Monocular standard automated perimetry was performed for both eyes by an experienced ophthalmic technician, with appropriate refractive correction. All visual fields were subsequently reviewed qualitatively by a board-certified ophthalmologist. Because two different test protocols were used across patients (SITA 24 − 2 in some and SITA 30 − 2 in others), quantitative visual field analysis was restricted to the Visual Field Index (VFI), a normalized global index expressed as a percentage of age-adjusted normal. The primary analyses relied on within-subject comparisons of VFI between the operated and contralateral eyes; other global indices such as mean deviation (MD) or pattern standard deviation (PSD), which are more sensitive to differences in test pattern, were not used for between-eye or between-patient comparisons. Abnormal visual fields were defined according to deviation from the manufacturer’s normative database. Reliability indices—including fixation losses, false positives, and false negatives—were reviewed for each examination. This approach allowed detection of both generalized and localized optic nerve dysfunction that might not be clinically evident.
3. Optical Coherence Tomography (OCT) – Retinal Nerve Fiber Layer (RNFL) Analysis
  Structural assessment of the optic nerve was performed using spectral-domain optical coherence tomography (SD-OCT) with the Heidelberg Spectralis^®^ system (Heidelberg Engineering, Germany) used for high-resolution RNFL segmentation. Peripapillary circular scans centered on the optic disc were acquired for both eyes using the device’s standard RNFL protocol.
  Quantitative analysis included average RNFL thickness and quadrant-specific values (superior, inferior, nasal, and temporal), reported in micrometers (µm). Scans with poor signal strength, motion artifacts, or other segmentation errors were excluded from the analysis. All OCT data were acquired by trained technicians and independently reviewed by an experienced ophthalmologist blinded to the surgical side.
  To identify iatrogenic subclinical structural damage retinal nerve fiber layer (RNFL) thickness was analyzed both as a global average and within individual quadrants (superior, inferior, nasal, and temporal). Measurements obtained from the operated side were compared with the corresponding values from the contralateral (non-operated) side. This intra-subject, quadrant-specific comparison allowed detection of localized RNFL thinning that may not be evident in global averages.
4. Visual Evoked Potentials (VEPs)
  Electrophysiological assessment of the anterior visual pathway was performed using pattern-reversal visual evoked potentials (VEPs). A high-contrast checkerboard pattern stimulus was presented on a monitor at a fixed distance under appropriate lighting conditions, and each eye was tested monocularly while the contralateral eye was occluded.
  Electrical responses were recorded via scalp electrodes placed at Oz (active), Fz (reference), and Fpz (ground). The primary parameters analyzed were P100 latency (in milliseconds) which reflect the electrophysiological conduction properties of the optic nerve and anterior visual pathway.
  All recordings were performed by trained technicians and independently reviewed by a certified neurophysiologist (clinical neurologist) who was fully blinded to the surgical details, including the side of surgery and the study hypothesis. The use of VEPs enabled objective quantification of conduction delays or transmission deficits that may not be detected through visual acuity, field testing, or structural imaging alone. P100 values taken from the surgical side were compared with values taken from the intact side visual pathway.

### Ethical aspects

This retrospective study was approved by the local institutional ethics committee, and the requirement for written informed consent for the retrospective chart review was waived. For the postoperative ophthalmologic and electrophysiological assessments performed at follow-up, all participants provided oral and written informed consent prior to testing. All data were anonymized before analysis, and the study was conducted in accordance with the Declaration of Helsinki (1964) and its subsequent revisions. Approval was granted by the Istanbul Medeniyet University Non-Interventional Health Research Ethics Committee (Document No. 2025/09–21).

### Statistical analysis

All statistical analyses were performed using IBM SPSS Statistics for Windows, Version 26.0 (IBM Corp., Armonk, NY, USA).

Data were tested for normality using both the Shapiro–Wilk test and the Kolmogorov–Smirnov test. These tests were used to check whether the data followed a normal distribution. Variables that were normally distributed were expressed as mean ± standard deviation (SD) and analyzed using parametric tests, while non-normally distributed variables were reported as median with interquartile range (IQR) and analyzed using non-parametric tests.

Comparisons between the operated and contralateral sides were conducted using the paired-samples t-test when the differences between paired outcome variables were normally distributed, and the Wilcoxon matched-pair signed-rank test was used for non-normally distributed differences. For paired analyses, the null hypothesis was that the mean difference between the operated and contralateral sides was equal to zero, indicating no significant difference.

For within-subject comparisons involving more than two related measurements, a one-way repeated measures analysis of variance (ANOVA) was used. When assumptions of sphericity were violated, the Greenhouse–Geisser correction was applied. Post-hoc pairwise comparisons were conducted with Bonferroni correction to adjust for multiple testing.

A p-value < 0.05 was considered statistically significant.

## Results

Between January 2023 and December 2024, all patients who were operated on for ruptured aneurysm, in whom extradural anterior clinoidectomy (EAC) was performed and who met the inclusion criteria, were included in the study, and a total of 17 patients were initially identified. One of these patients had moderate bilateral loss of visual acuity, which was also confirmed by RNFL measurements. Detailed ophthalmological examination was performed because of the bilateral involvement that was inconsistent with the cranial lesion and revealed a diagnosis of terminal glaucoma. This patient was subsequently excluded from the study. Consequently, 16 patients who fully met the inclusion and exclusion criteria constituted the final study group.

The mean age of the patients was 50.4 ± 15.4 years (range, 25–81 years), and the median age was 50.5. Of these cases, 10 (62.5%) were male and 6 (37.5%) were female. The surgical side was right in 10 cases (62.5%) and left in 6 cases (37.5%).

When the distribution of pathologies among the included patients was examined, the most common lesion was an anterior communicating artery (ACom) aneurysm, observed in 13 cases (81.3%). A posterior communicating artery (PCom) aneurysm was detected in two cases (12.5%), and a communicating segment internal carotid artery (ICA) aneurysm in one case (6.3%). Sex, surgical side, and pathology distributions are summarized in Table 1.

**Table 1:** Demographic and clinical characteristics of the patients (*n* = 16). This table summarizes baseline patient characteristics, including age, sex, surgical side, and aneurysm pathology. Overall, the cohort was predominantly male, with most procedures performed on the right side and the majority of cases involving anterior communicating artery aneurysms

Variable	Value
Side	Left side: 6 (37.5%)
	Right side: 10 (62.5%)
Pathology	AComA aneurysm: 13 (81.3%)
	ICA aneurysm: 1 (6.3%)
	PComA aneurysm: 2 (12.5%)
Sex*	Male: 10 (62.5%)
	Female: 6 (37.5%)

### Visual acuity

Best-corrected visual acuity was preserved in all patients. Bilateral full visual acuity (1.0/1.0) was observed in 15 of the 16 patients. In one patient, a mild asymmetry was noted, with 0.7 in the operated eye and 0.9 in the contralateral eye. There was no clinically significant reduction of visual acuity at the operated-sides of the patients. Because of the limited variability in the data, no further statistical analysis was performed for this parameter.

### Retinal nerve fiber layer (RNFL) thickness

The mean global RNFL thickness was 99.19 ± 18.57 μm in the contralateral eye and 95.81 ± 12.73 μm in the ipsilateral eye. Although the values were slightly higher on the contralateral side, the difference was not statistically significant (mean difference 3.38 μm, 95% CI: − 2.37 to 9.12; *p* = 0.230).

**Fig. 2:**
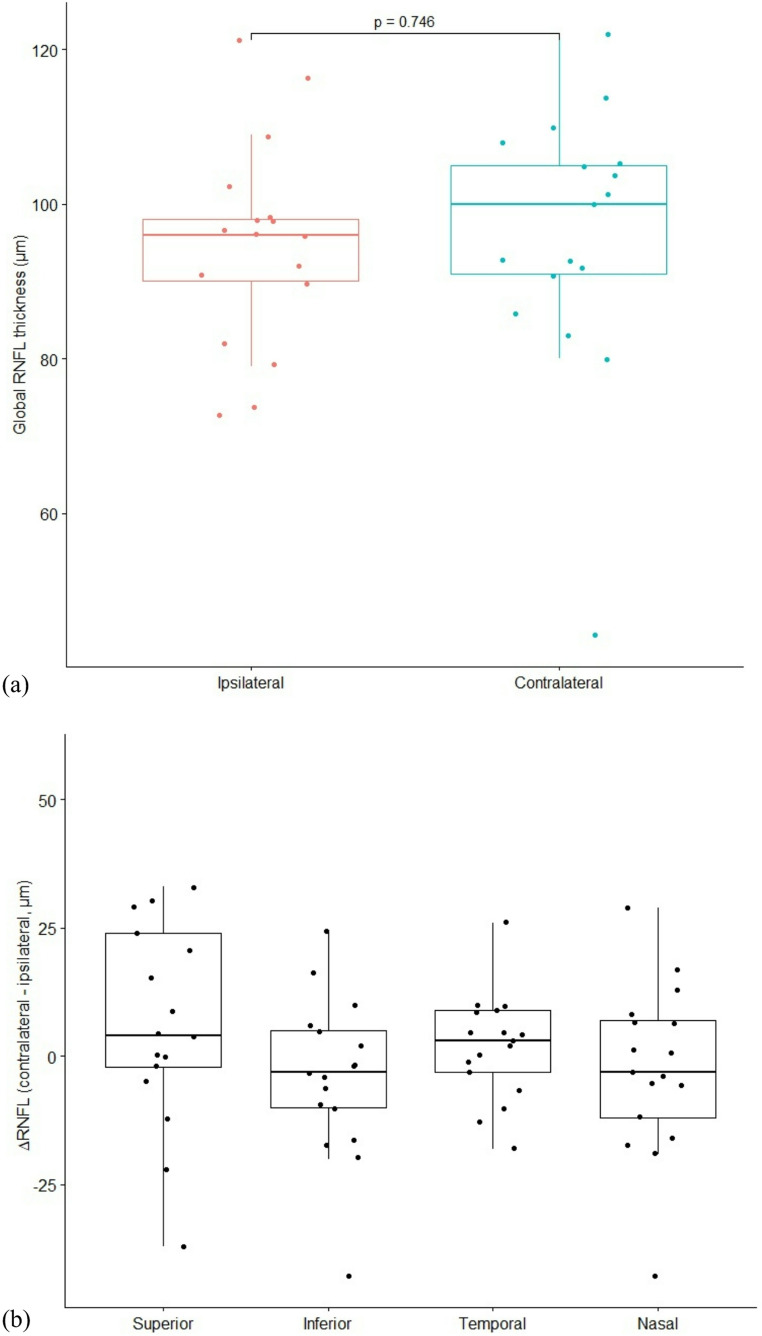
(**a**) Global RNFL thickness in both eyes after extradural anterior clinoidectomy. (**b**) Distribution of ΔRNFL differences between the ipsilateral and contralateral eyes by quadrant

These findings indicate that extradural anterior clinoidectomy does not cause a clinically meaningful inter-eye difference in the global RNFL thickness (Fig. 2a).

In the quadrant-based analysis, the inter-eye difference (ΔRNFL) was calculated using the following formula.

$$\:{\Delta\:}RNF{L}_{\left\{Quadrant\right\}}=\:RNF{L}_{\left\{Contralateral\right\}}-\:RNF{L}_{\left\{Ipsilateral\right\}}$$

Repeated-measures ANOVA performed on quadrant-specific ΔRNFL (contralateral – ipsilateral) values demonstrated a significant effect of quadrant (F(3,48) = 4.176, *p* = 0.01, partial η² = 0.207). This finding indicates that inter-eye differences varied across quadrants. The mean ΔRNFL values were + 3.06 ± 9.29 μm in the temporal quadrant, 0.00 ± 13.13 μm in the nasal quadrant, + 12.81 ± 23.30 μm in the superior quadrant, and − 1.63 ± 11.97 μm in the inferior quadrant. Although the largest positive difference was observed in the superior quadrant, Bonferroni-corrected pairwise comparisons revealed no significant differences between any quadrant pairs (all *p* > 0.05) (Table 2; Fig. 2b).

**Table 2:** Comparison of global and quadrant-based RNFL thicknesses between ipsilateral and contralateral eyes. This table summarizes postoperative OCT-derived RNFL thickness in the operated (ipsilateral) and contralateral eyes, including global and quadrant values (temporal, nasal, superior, inferior). Global RNFL was compared with paired testing, and quadrant-based ΔRNFL was assessed using repeated-measures ANOVA

Region	Ipsilateral (Mean ± SD, µm)	Contralateral (Mean ± SD, µm)	Mean Difference (Mean ± SD, µm)	*p*-value
Global	95.81 ± 12.73	99.19 ± 18.57	3.38 ± 10.79	0.230¹
Temporal	67.13 ± 11.89	70.19 ± 13.26	3.06 ± 9.29	0.011²
Nasal	78.00 ± 16.29	78.00 ± 22.03	0.00 ± 13.13	
Superior	110.38 ± 26.93	123.19 ± 17.23	12.81 ± 23.30	
Inferior	127.38 ± 13.71	125.75 ± 15.74	–1.63 ± 11.97	

¹ p-value corresponds to the paired t-test result

² p-value corresponds to the repeated-measures ANOVA result

The superior quadrant demonstrated the greatest inter-eye variability (SD in the range of ~ 23–26 µ and a numerically greater thinning in the operated eye (mean difference ≈ 12–13 μm). Although this difference did not reach statistical significance after correction for multiple comparisons, the relatively small sample size and inherent variability of RNFL measurements limit the statistical power of the study, and a type II error cannot be excluded. Given its anatomical proximity to the surgical corridor during optic canal unroofing, the superior quadrant may represent a region of relative vulnerability to minimal traction or manipulation. Therefore, this finding should be interpreted cautiously and viewed as hypothesis-generating rather than definitive evidence of absence of structural impact. Importantly, no corresponding consistent functional or electrophysiological deterioration was detected across the cohort.

In the nasal quadrant, no inter-eye difference was observed (mean ΔRNFL ≈ 0.0 μm), and the variability was relatively low. This suggests that nasal RNFL is more stable than the temporal and superior quadrants and may be less affected because of its relative distance from the surgical corridor.

### Perimetry and visual field index (VFI)

In the ipsilateral eye, the median VFI was 97 (IQR = 7), and in the contralateral eye it was 97 (IQR = 6). Because the data in both eyes were not normally distributed, parametric tests were not used. Comparison with the Wilcoxon signed-rank test showed no statistically significant difference (*p* = 0.7). Although VFI values dropped to the mid-40s in two patients, the corresponding structural (RNFL), electrophysiological (VEP), and visual acuity tests were normal. This discrepancy suggests that the abnormal VFI values may reflect test unreliability related to poor patient cooperation or false-positive responses rather than true optic nerve dysfunction. Therefore, in some patients especially in the elderly population, automated perimetry may not yield reliable results, and the findings should be interpreted with caution and correlated with other testing modalities.

**Fig. 3:**
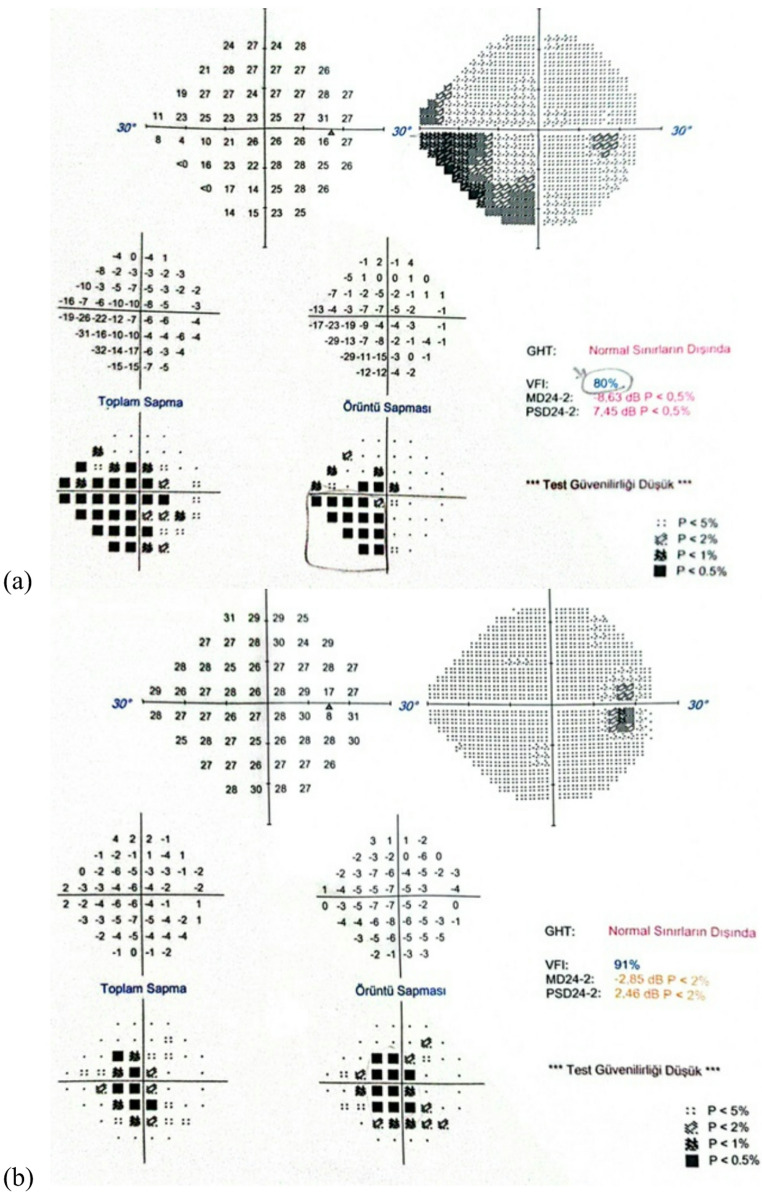
(**a**) Automated perimetry report showing inferior nasal quadrantanopia on the operated side in a patient who underwent surgery for a communicating segment ICA aneurysm. (**b**) Automated perimetry report from one of two patients in whom asymptomatic enlargement of the blind spot was detected on the operated side after extradural anterior clinoidectomy

In addition, in one patient who was operated on for a communicating segment ICA aneurysm, postoperative ipsilateral inferior nasal partial quadrantanopia was detected, which can also be attributed to possible iatrogenic optic nerve manipulation (Fig. 3a).

In addition, a mild, subclinical enlargement of the blind spot was observed in two patients (Fig. 3b). Enlargement of the blind spot typically reflects subtle peripapillary dysfunction or changes at the level of the optic nerve head. In the context of extradural anterior clinoidectomy, a possible mechanism may involve traction or manipulation at the dural sheath as the optic canal roof is removed and the anterior clinoid process is detached. Such maneuvering could theoretically transmit minimal mechanical stress to the peripapillary region or alter microvascular perfusion at the optic nerve head. Importantly, these changes were not accompanied by consistent RNFL thinning or significant VEP latency prolongation, suggesting that any structural or electrophysiological impact, if present, was subtle and subclinical. Nevertheless, this observation warrants cautious interpretation and further investigation in larger cohorts.

### Electrophysiological evaluation and P100 latency

**Fig. 4:**
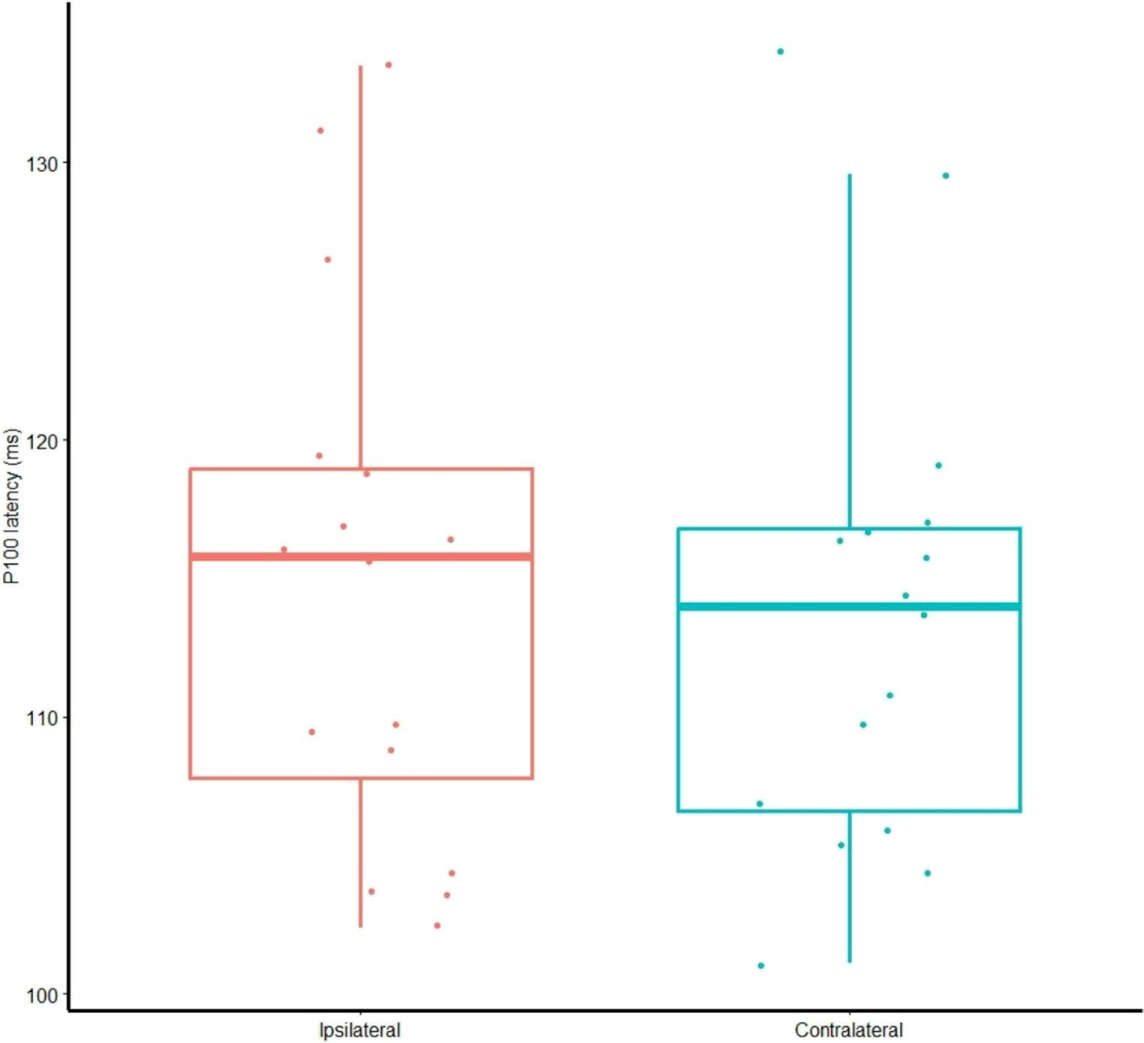
Distribution of P100 latency in the ipsilateral and contralateral eyes

In visual evoked potential (VEP) assessment, P100 latency was 114.8 ± 9.7 ms in the ipsilateral eye and 113.8 ± 8.9 ms in the contralateral eye. Although the raw data were not normally distributed, the distribution of the differences between sides was normal, so a parametric test was applied. A paired t-test revealed no statistically significant difference (mean difference = 0.99 ms; 95% CI − 0.67 to 2.64; *p* = 0.223) (Fig. 4).

Mean values were determined by calculating the P100 latencies obtained from the A1 and B1 waves.

## Discussion

In this study, we evaluated the functional, structural, and electrophysiological effects of extradural anterior clinoidectomy in patients without preoperative optic nerve compression. While no statistically significant inter-eye differences were observed across VFI, RNFL thickness, or P100 latency, postoperative visual field changes occurred in a subset of patients, and a trend toward ipsilateral superior RNFL thinning was noted.

The observed trend toward superior quadrant RNFL thinning, although not statistically significant, may reflect the increased susceptibility of optic nerve fibers within the optic canal during surgical exposure and deroofing. During extradural anterior clinoidectomy, manipulation at the level of the optic canal roof may induce subtle mechanical traction on the superior aspect of the optic nerve. Alternatively, particularly in aneurysm cases, microvascular compromise involving perforating vessels supplying the superior nerve fibers may contribute to these subclinical structural changes. These mechanisms are consistent with the close anatomical relationship between the superior surface of the optic nerve and the optic canal roof.

Similarly, although the visual field index did not differ significantly between eyes, visual field changes were observed in a small number of patients, including two cases of subclinical blind spot enlargement and one case of ipsilateral inferior nasal quadrantanopia. Blind spot enlargement may reflect subtle peripapillary dysfunction at the level of the optic nerve head, potentially related to mild traction on the optic nerve sheath during optic canal unroofing or clinoid removal, with transient effects on axoplasmic flow or microvascular perfusion. These findings were not accompanied by consistent RNFL thinning or significant VEP latency changes. However, the occurrence of quadrantanopia suggests that focal visual pathway disturbances may occur in individual cases. Therefore, subtle adverse effects of clinoidectomy on the optic nerve cannot be completely excluded.

To our knowledge, this is the first study to evaluate the isolated effect of extradural anterior clinoidectomy on optic nerve integrity using multimodal testing in patients without preoperative optic nerve compression. Previous studies have primarily focused on cases in which optic nerve dysfunction was already present due to tumor invasion or aneurysmal compression, making it difficult to isolate the independent effect of clinoidectomy itself. By excluding patients with optic canal invasion, optic nerve compression, or preoperative visual deficits, our study was designed to evaluate the procedure in a setting where the optic nerve was anatomically uncompromised.

Several mechanisms of potential optic nerve injury during clinoidectomy have been proposed, including direct mechanical trauma, traction transmitted during bone removal, thermal injury from drilling, and vascular compromise of the ophthalmic artery or its branches [11]. However, in most previously published series, visual outcomes have been influenced primarily by the underlying pathology—such as paraclinoid aneurysms or clinoidal/parasellar meningiomas—rather than by the clinoidectomy procedure itself [3, 12]. For example, Lehmberg et al. reported improvement in visual function in 79% of patients undergoing optic nerve decompression for tumors involving the optic canal while only small proportion of patients experienced postoperative deterioration. In this series, although optic nerve decompression and/or anterior clinoidectomy was performed in a total of 34 patients, the authors stated that both extradural and intradural techniques were used in different patients but did not provide detailed information on the distribution of each approach. Furthermore, because these cases involved preexisting optic nerve compression, it is difficult to determine the independent effect of the clinoidectomy procedure itself [12].

According to the literature, in cases with marked optic nerve compression or optic canal invasion, intradural anterior clinoidectomy allows early release of the falciform ligament, producing an immediate partial decompression of the optic nerve. This early release may reduce the mechanical impact of subsequent manipulations on the optic nerve during bony decompression and tumor resection. This point is further supported by the series of Paulo Henrique Pires de Aguiar et al. [4], who reported higher rates of vision-related complications in patients undergoing extradural clinoidectomy compared with an intradural approach in a cohort of 36 paraclinoid aneurysm cases.

Our study therefore addresses a different clinical scenario—patients without optic nerve compression in whom EAC was performed to improve surgical exposure. In our cohort, extradural anterior clinoidectomy is selectively employed in ruptured aneurysm cases with limited cisternal access, particularly in anterior communicating artery aneurysms; however, this approach represents a surgeon-specific preference and is not considered standard practice for routine ACom aneurysm surgery.

In our institutional practice, extradural anterior clinoidectomy is selectively employed in ruptured aneurysm cases when early cisternal access is limited due to brain tightness. Removal of the anterior clinoid process expands the surgical corridor to the optico-carotid cisterns, facilitating early cerebrospinal fluid release and reducing the need for brain retraction. Importantly, in the present series optic canal unroofing was performed only to detach the medial attachment of the anterior clinoid process and facilitate its removal, rather than to decompress an already compressed optic nerve. Consequently, the extent of manipulation around the optic nerve was relatively limited. However, particularly in cases of anterior communicating artery aneurysms, this approach reflects a surgeon-specific or institutional preference rather than standard practice for routine ACom aneurysm surgery.

The safety of drill-free clinoidectomy techniques has also been suggested in previous studies. Niibo et al. [14] compared drill-based and no-drill anterior clinoidectomy in patients undergoing surgery for paraclinoid aneurysms and reported permanent visual impairment in 21.9% of patients in the drill group, whereas no permanent deficits occurred in the no-drill group. Although these findings support the potential safety advantages of the drill-free technique, several methodological limitations were present in that study, including unequal group sizes, lack of randomization, absence of structural imaging modalities like OCT, and incomplete description of visual field testing methodology. In addition, heterogeneity in aneurysm characteristics may have introduced additional confounding factors. Importantly, these limitations may have reduced the sensitivity of the study to detect subtle or subclinical structural optic nerve changes, such as mild RNFL thinning. In addition, data on optic nerve outcomes specifically related to no-drill techniques remain limited. Nevertheless, the favorable outcomes reported after adoption of the no-drill technique support the concept that minimizing mechanical and thermal stress during clinoidectomy may reduce the risk of overt optic nerve injury. In this context, our study provides additional insight by incorporating a multimodal assessment of postoperative optic nerve function. Automated perimetry detects functional visual field changes, OCT-derived RNFL measurements assess structural integrity, and visual evoked potentials evaluate electrophysiological conduction. The combined use of these complementary modalities increases sensitivity for detecting subtle optic nerve dysfunction beyond visual acuity alone.

To further enhance interpretability, our study design was intentionally restricted to a homogeneous clinical scenario. The present study was intentionally restricted to ruptured aneurysm cases to maintain surgical and intraoperative homogeneity. In our institutional practice, extradural anterior clinoidectomy is primarily used in cases of ruptured aneurysms with brain swelling or tight cisterns. Including unruptured aneurysms would introduce heterogeneity in surgical indication, intraoperative conditions, and degree of manipulation, thereby complicating interpretation of the isolated effect of the clinoidectomy procedure.

Moreover, internal carotid artery (ICA) aneurysms that could compress the optic nerve or require mobilization of the optic nerve for clipping or for obtaining proximal control at the clinoid segment were not included in our study. Such pathologies may themselves predispose patients to traumatic or ischemic optic neuropathy due to direct compression or surgical manipulation of the optic apparatus. By excluding these lesions, the present study aimed to minimize confounding factors and better isolate the potential effect of the clinoidectomy procedure itself.

Apart from these considerations, our study also has several strengths. The comparison of ipsilateral and contralateral eyes within the same patient allowed each individual to serve as their own internal control, thereby minimizing inter-individual variability. This within-subject design reduces the influence of systemic factors and testing conditions and increases the reliability of the findings despite the relatively small sample size. However, it should be acknowledged that the inclusion of preoperative baseline measurements would have provided a more robust reference for detecting subtle postoperative changes and remains a limitation of the present study.

Nevertheless, several limitations of the study should be acknowledged. First, the study was conducted at a single center and included a relatively small number of patients, limiting statistical power and generalizability. Second, all procedures were performed by a single surgeon, which reduces variability in surgical technique but limits assessment of surgeon-related variability. Third, preoperative multimodal visual testing was not available; while such baseline assessment would have provided a more robust reference for detecting subtle postoperative changes, its reliable acquisition in the acute ruptured aneurysm setting is often limited by patient condition and cooperation. Therefore, the analysis relied on postoperative inter-eye comparisons as a pragmatic internal control, although it does not replace true baseline measurements.

Furthermore, all patients presented with aneurysmal subarachnoid hemorrhage, which may introduce systemic and intracranial factors affecting both eyes, such as transient intracranial pressure elevations or ocular complications (e.g., Terson syndrome). As a result, the contralateral eye serves as an internal comparator rather than a truly unaffected control. In addition, microvascular disturbances or vasospasm related to basal cistern dissection may influence optic pathway function. Although most postoperative findings were within normal ranges, these factors should be considered when interpreting the results.

Future studies involving larger multicenter cohorts and standardized pre- and postoperative visual assessments will be important to further clarify whether subtle structural or microvascular changes occur following anterior clinoidectomy. Advanced imaging modalities such as optical coherence tomography angiography (OCTA) or diffusion tensor imaging (DTI) may provide additional insight into microvascular perfusion and axonal integrity that cannot be detected by conventional RNFL thickness measurements alone.

## Conclusion

Although extradural anterior clinoidectomy was not associated with statistically significant or consistent deterioration in optic nerve function across the cohort, postoperative visual field changes were observed in 3 of 16 patients (18.75%), including one case of clinically evident quadrantanopia. In addition, a trend toward ipsilateral RNFL thinning in the superior quadrant was noted, although this did not reach statistical significance. These findings suggest that while the procedure appears largely safe in patients without preoperative optic nerve compression, subtle structural or functional alterations may occur and should be interpreted with caution, particularly in small cohorts. Further validation in larger, adequately powered studies is warranted.

## Data Availability

The datasets generated and analyzed during the current study are not publicly available in accordance with institutional policies and ethics committee approval conditions.
